# Systematic evaluation on the physicochemical characteristics of a series polysaccharides extracted from different edible lilies by ultrasound and subcritical water

**DOI:** 10.3389/fnut.2022.998942

**Published:** 2022-09-20

**Authors:** Zihan Song, Yanli Zhang, Yulin Luo, Yongrui Ti, Weizhen Wang, Yuqian Ban, Yuchao Tang, Yuqing Hou, Leifeng Xu, Jun Ming, Panpan Yang

**Affiliations:** ^1^Institute of Vegetables and Flowers, Chinese Academy of Agricultural Sciences, Beijing, China; ^2^College of Horticulture, Shanxi Agricultural University, Taigu, China; ^3^School of Agriculture, Yunnan University, Kunming, China

**Keywords:** lily, polysaccharides, subcritical water, ultrasonic-assisted extraction, physicochemical properties

## Abstract

A series polysaccharide samples extracted from three edible lilies (*Lilium davidii* var. *willmottiae*, *Lilium brownii* var. *viridulum*, and *Lilium lancifolium*) by subcritical water and ultrasound-assisted extraction were systematically compared. The results showed that extraction method was a more important factor than lily species. Subcritical water extracted lily polysaccharides (S-LP) with higher yield, molecular weight, neutral glucose and uronic acid content as well as apparent viscosity. Ultrasound-assisted extracted lily polysaccharides (U-LP) with higher reducing sugars and protein content. Moreover, due to the degradation of glycosidic bonds, ultrasonic extraction was easier to obtain lower molecular weight polysaccharides. In addition, the extraction method significantly affected the monosaccharide proportion of polysaccharides, but had no effect on type. Glucose was the main component in S-LP, and glucose and mannose were the main components in U-LP. The micromorphology of different polysaccharide samples was similar, and the scanning electron microscope (SEM) images showed regular/irregular particle clusters with different particle sizes. Overall, the relationships between extraction methods, lily species and polysaccharide properties were preliminarily elucidated, providing a reference for the targeted extraction of specific lily polysaccharides (LP).

## Introduction

Lily belongs to the genus *Lilium* of the family Liliaceae. It is not only ornamental, but also has edible and medicinal value. In China, *Lilium davidii* var. *willmottiae*, *Lilium brownii* var. *viridulum* and *Lilium lancifolium* are the largest planting species and known as the “three edible lilies.” Bulb, as the important edible part of lily, is not only a good source of nutrient substances including starch, protein and dietary fiber, but also contains a variety of bioactive substances, such as polysaccharides, saponin, and alkaloids (1, 2). Lily polysaccharides (LP) show various biological capacities including such as anti-tumor (3), anti-oxidation (4) and immunomodulatory effects (5). At present, the studies on Lily polysaccharides mainly focus on functional and structural analysis (6–8). There are few studies on the chemical composition, monosaccharide composition and rheology of different lily species on polysaccharides.

There are numerous extraction methods which have been explored for the preparation of polysaccharides, and different methods have their own advantages and disadvantages (9). Ultrasonic-assisted extraction (UAE) is a common method to extract lily polysaccharides (10). The technique extracts polysaccharides through cavity effect, thermal effect and mechanical effect produced by ultrasound. However, temperature rise during extraction is difficult to monitor, which may lead to low reproducibility of results (10). In addition, ultrasound treatment is easy to generate polysaccharides with low molecular weight (Mw) and can degrade part of the polysaccharides (11, 12).

Subcritical water (SW) is increasingly used for the extraction of polysaccharides and other bioactive substances in recent years due to its advantages of energy saving, environmental protection and high extraction efficiency (13). However, no systematic evaluation on lily polysaccharides extracted by subcritical water has been reported. Subcritical water is a liquid hot water with a temperature above the boiling point (100°C) and below the critical point (374°C) (14). In the subcritical state, the dielectric constant, polarity and viscosity of water decrease and the ionization constant increases, which can improve the heat transfer process and increase the extraction efficiency of polysaccharides (15–17). However, subcritical water is not suitable for industrial production due to its cumbersome operation.

To the best of our knowledge, there has not been systematic evaluation for the relationship between the extraction methods and different lily species on physicochemical properties of polysaccharides. In this study, we collected the bulbs of *Lilium davidii* var. *willmottiae*, *Lilium brownii* var. *Viridulu*m, and *Lilium lancifolium* as raw materials to prepare polysaccharides. And systematically compared the polysaccharide samples obtained by subcritical water and ultrasonic-assisted extraction for the first time. By comparing the yield, physicochemical, spectral and rheological properties, the regularity of LP prepared by two extraction methods was clarified, and the differences of physicochemical indexes of different lily species were observed. This study will provide some references for the extraction method and variety selection of lily polysaccharides based on different research purposes.

## Materials and methods

### Materials and reagents

Fresh bulbs from three edible lily species, *Lilium davidii* var. *willmottiae*, *Lilium brownii* var. *viridulum*, and *Lilium lancifolium*. were collected from Lanzhou City, Gansu Province, Longhui County, Hunan Province, and Longshan County, Hunan Province, China, respectively. Monosaccharides (fucose, rhamnose, arabinose, galactose, glucose, xylose, mannose, fructose, ribose, galacturonic acid, glucuronic acid, mannuronic acid, and guluronic acid) were purchased from Sigma Company (St. Louis, MO, USA). A series of polyethylene glycol standards were purchased from PSS Polymer Standards Service GmbH. Other commonly used reagents were analytically pure and purchased from local suppliers.

### The polysaccharide extraction

#### Subcritical water extraction

Briefly, subcritical water extraction was carried out in a reactor equipped with a heating system and a control system. The dry powder of lily bulb and distilled water were added in a 1 L reaction kettle with a ratio of 1:15, and extracted for 10 min when the temperature raised to 150°C. After the reaction, the mixture was centrifuged at 6,000 rpm for 10 min to obtain the supernatant. The supernatant was added with anhydrous ethanol (>99.5%) and precipitated at 4°C for 12 h. The precipitates were collected by centrifugation again and freeze-dried to obtain three kinds of lily polysaccharides (9, 18).

#### Ultrasonic-assisted extraction

Ultrasonic processor was used for ultrasonic-assisted extraction. The ratio of material to liquid was 1:20 and the reaction condition was 65°C for 20 min. The three kinds of lily polysaccharides were obtained by the same process of alcohol precipitation, centrifugation and freeze-dried described above. The lily polysaccharides yield (%) was calculated as follows:


(1) LilypolysaccharidesYield(wt%)=W/2W×1100

Where: W_1_ was the residue dried (g) weight of lily bulb, W_2_ was the weight of the extracted lily polysaccharides (g) (19).

### Physiochemical properties of lily polysaccharides

#### Chemical composition

The content of neutral sugar and uronic acid were determined by phenol-sulfuric acid method and carbazole method, respectively, using glucose and D-galacturonic acid as standard (20, 21). The content of reducing sugar was determined using the 3,5-dinitrosalicylic acid (DNS) method (22). Protein content was determined by Bradford method with bovine serum albumin as standard (23).

#### Molecular weight

The Molecular weight (Mw) of LP samples were detected by gel permeation chromatography (GPC) (Agilent1260, California, USA) (9). 40 μl LP solution (1 mg/ml) was dissolved in sodium azide and sodium acetate was added into the chromatographic system. Waters Ultrahydrogel TM120 TM250 TM500 water-soluble gel column (7.8 × 300 mm triple column series) was used for the determination. Forty microliters LP solution (1 mg/ml) was dissolved in sodium azide and sodium acetate was added into the chromatographic system. The analyzed at 30°C at a flow rate of 1 ml/min. Polyethylene glycol standards with different molecular weights (1,960; 4,290; 7,130; 12,600; 20,600; 25,300; 44,000; 78,300; 152,000; 326,000) were administered for calibration.

#### Monosaccharide composition

The monosaccharide composition of lily polysaccharides was determined by high performance anion exchange chromatography (HPAEC) (24). Five milligrams crude LP samples were hydrolyzed in sealed ampoule with trifluoroacetic acid (2 M) at 121°C for 2 h. Then the samples were dried by nitrogen and washed by methanol, and the procedure was repeated three times. The residue was re-dissolved in deionized water and filtered by 0.22 μm microporous membrane for measurement. The chromatographic system was a Thermo ICS5000 ion chromatography system (ICS5000, Thermo Fisher Scientific, MA, USA). Chromatographic conditions included a CarboPac PA-20 Ionic Exchange column (3 × 150 mm) and a pulsed Amperometric detector (PAD; Dionex ICS 5000 system; Shanghai, China). The loading volume was 5 μl, and the flow rate was 0.5 ml/min. The solvent system included solvent system A: (ddH_2_O), solvent system B: (0.1 M NaOH), solvent system C: (0.1 M NaOH, 0.2 M NaAc); The gradient program was shown in Table 1. In addition, the 11 monosaccharide standards needed in this experiment were purchased from Sigma Company (St. Louis, MO, USA), and the information of standard products is shown in Table 2.

**TABLE 1:** Gradient program for high performance anion exchange chromatography (HPAEC).

Time (min)	A (%)	B (%)	C (%)
0	95	5	0
26	85	5	10
42	85	5	10
42.1	60	0	40
52	60	40	0
52.1	95	5	0
60	95	5	0

**TABLE 2:** Information on monosaccharide standards.

Name	Abbreviation	CAS number	Molecular formula
Fucose	Fuc	2438-80-4	C_6_H_12_O_5_
Rhamnose	Rha	10030-85-0	C_6_H_14_O_6_
Arabinose	Ara	5328-37-0	C_5_H_10_O_5_
Galactose	Gal	26566-61-0	C_6_H_12_O_6_
Glucose	Glc	50-99-7	C_6_H_12_O_6_
Xylose	Xyl	58-86-6	C_5_H_10_O_5_
Mannose	Man	3458-28-4	C_6_H_14_O_6_
Fructose	Fru	57-48-7	C_6_H_12_O_6_
Ribose	Rib	50-69-1	C_5_H_10_O_5_
Galacturonic acid	Gal-UA	14982-50-4	C_6_H_10_O_7_
Glucuronic acid	Glc-UA	6556-12-3	C_6_H_10_O_7_
Mannuronic acid	Man-UA	6814-36-4	C_6_H_10_O_7_
Guluronic acid	Gul-UA	15769-56-9	C_6_H_10_O_7_

### Spectrometric analysis of lily polysaccharides

#### Ultraviolet–visible spectrum

The ultraviolet–visible (UV) spectrum of LP samples were determined with ultraviolet–visible spectroscopy (Shimadzu UV-2600, Tokyo, Japan). LP samples were prepared as 0.5 mg/ml polysaccharides solution with distilled water, and the UV absorption curves of the solution were recorded in the wavelength range of 200–900 nm (25).

#### Fourier transform infrared spectrum

The infrared spectrum characteristics of LP samples were determined by Fourier infrared spectrophotometer (Thermo Nicolet NEXUS870, Massachusetts, USA) within the wavenumber range of 4,000–500 cm^–1^. And the samples were prepared by KBr Tablet pressing method (18, 26).

### Scanning electron microscope analysis of lily polysaccharides

Field emission scanning electron microscopy (Hitachi S-4800, Tokyo, Japan) was used to observe the apparent morphology of LP obtained by different extraction methods. The polysaccharide samples were fixed on the carrier platform and sprayed with gold powder. The magnification used includes 25, 50, 250, and 500× (27).

### Rheological analysis

Hybrid Rheometer (MCR302, Anton Paar, Austria) was used to measure the static shear rheology properties of LP fractions (0.02 g/ml). Flow curves were measured under an increasing shear rate region (0.1–100 s^–1^) with a 40 mm parallel plate geometry with a gap size of 1.0 mm, and the angular frequency of 10 rad/s and the strain of 1% at 25°C were used (28).

### Statistical analysis

All data reported were averages of three replicates. And ANOVA and Duncan’s multiple range test were used to analyze the differences between data (*P* < 0.05). Finally, Origin software (2021 version) was used to draw the resulting image.

## Results and discussion

### The yield of lily polysaccharides

The yield is a crucial parameter to evaluate the extraction efficiency of subcritical and ultrasound-assisted extraction methods. In this study, the yield of LP extracted by SW was higher than 34.73% and ranged from 34.73 to 54.91%, while the yield of polysaccharides extracted by UAE was lower, ranging from 3.69 to 7.69% (Table 3). The yield of ultrasound-assisted extracted lily polysaccharides (U-LP) was less than 10%, which is similar to that reported by Xie et al. They used ultrasound-assisted different enzymes to extract polysaccharides of *Lilium davidii* var. *willmottiae* (6).

**TABLE 3:** The yield and molecular weight of lily polysaccharides.

Extraction method	Polysaccharide type	Yield (%)	Molecular weight (kDa)
S-LP	LDWP	34.73 ± 0.87^Cc^	322
	LBVP	54.91 ± 0.63^Aa^	333
	LLAP	49.32 ± 2.87^Bb^	389
U-LP	LDWP	7.69 ± 1.82^Dd^	3
	LBVP	3.69 ± 0.69^Ee^	53
	LLAP	4.53 ± 0.23^Ee^	26

The differences between the data can be represented by uppercase (*P* < 0.01) and/or lowercase letters (*P* < 0.05).

Obviously subcritical water method is a more suitable method to extract the high yield of LP. This may be attributed to the following two aspects: on the one hand, decreasing polarity of subcritical water dissolved polysaccharides contained in lily more easily and quickly. Also, the low density and viscosity of subcritical water contributed to the solubilization of polysaccharides (29–32). On the other hand, in the subcritical state, the ionization degree of aqueous solvent increased, and the high concentration of hydrogen and hydroxide ions could be used as acid-base catalysts to improve the yield of acidic polysaccharides (15, 33).

As shown in Table 2, the yield of LP was significantly different among different species (*p* < 0.05). Under the subcritical water extraction, the yield of *Lilium brownii* var. *viridulum* polysaccharides (LBVP) was the highest. Concerning the ultrasound-assisted extraction, the yield of *Lilium davidii* var. *willmottiae* polysaccharides (LDWP) was the highest, while the yields of LBVP and *Lilium lancifolium* polysaccharides (LLAP) were similar. The results indicate that subcritical water extraction had great potential in increasing the yield of lily polysaccharides. Moreover, *Lilium brownii* var. *viridulum* was a better species for the preparation of lily polysaccharides.

### Chemical composition of lily polysaccharides

**FIGURE 1:**
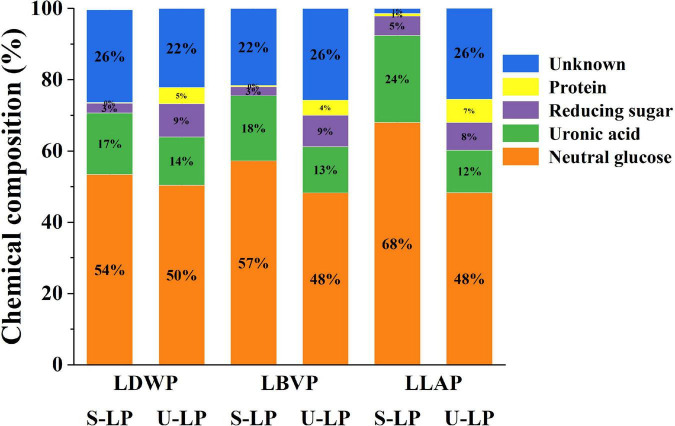
The chemical composition (%) of three lily polysaccharides extracted by different extraction methods.

The effects of two extraction methods on the chemical composition of lily polysaccharides were studied by anthrone-sulfuric acid method, carbazole method, DNS method, and Bradford method. The specific chemical compositions of crude lily polysaccharides were shown in Figure 1. The results showed that the extraction method had a significant effect on the chemical composition of LP. In addition, the proportions of neutral sugar, acid sugar, reducing sugar, and protein in different LP were different.

#### Neutral sugar

Neutral sugar content in crude polysaccharides of lily was shown in orange in Figure 1. Compared with ultrasound-assisted extraction, subcritical water extraction increased neutral sugar content by 3.35–19.70%. In addition, with the same extraction method, the neutral sugar content in lily polysaccharides of different species was also different. The neutral sugar content of LLAP was the highest in subcritical water extraction, while the LDWP was the highest in ultrasound-assisted extraction. Huang et al. also reported the difference of neutral sugar content in different LP (*Lilium lancifolium*, *Lilium davidii* var. *Unicolor*, and *Lilium brownii* var. *viridulum* Baker) with hot water extraction (32). The results showed that subcritical water extraction and *Lilium lancifolium* were better choices for obtaining high neutral sugar content.

#### Uronic acid

Uronic acids are considered to have enormous potential in the cosmetic, pharmaceutical and food industries (34). The content of uronic acid in crude LP showed the same trend as neutral sugar content (The green part of Figure 1). In other words, the content of uronic acid in crude LP obtained by subcritical water treatment was higher, ranged from 17.32 to 26.24%. And the proportion of uronic acid in LP obtained by ultrasonic-assisted extraction was less than 14%. Under the condition of subcritical water, the high concentration of hydronium ion promoted the hydrolysis of pectin in lily bulb to uronic acid. It has been reported that the highest yield of uronic acid can reach 79.7 g kg^–1^ with subcritical water hydrolysis of pectin rich biomass (31).

In addition, the content of uronic acid was also affected by lily species. This may be due to the difference in pectin content in lily bulbs of different species (35). In conclusion, the subcritical water extraction combined with the species of *Lilium lancifolium* Thunb was the best choice for obtaining high uronic acid content.

#### Reducing sugar

The reducing sugar content was shown in purple in Figure 1. The reducing sugar content of crude polysaccharides extracted from subcritical water ranged from 2.53 to 5.00%, while that of ultrasonic assisted extraction ranged from 7.08 to 9.36%. Obviously, compared with the differences of lily species, the extraction method was a more important reason for the changes in reducing sugar content. Reducing sugars were known to come from the breakdown of complex carbohydrates. Ismadji et al. reported that high temperature (120–160°C) and long reaction time (3–5 h) contribute to the breakdown of carbohydrates (36). Although the SW method in this study was carried out at high temperature, the reaction time was short (15 min) and only a small amount of carbohydrates could be decomposed. Therefore, the reducing sugar proportion of crude polysaccharides extracted from subcritical water was relatively low.

#### Protein

The protein content of crude polysaccharides was shown in yellow in Figure 1. The protein content of crude polysaccharides extracted by subcritical water extraction was less than 1%, and the protein content of S-LDWP was the lowest, as low as 0.29%. The protein content of crude polysaccharides obtained by ultrasonically assisted extraction ranged from 4.26 to 6.55%. The lower protein content indicated that the two extraction methods were able to extract polysaccharides from different lily species with higher purity, and subcritical water had higher extraction selectivity for polysaccharides.

In addition, with the exception of neutral sugars, acidic sugars, reducing sugars, and proteins, all samples also include some unknown composition, which may be composed of ash, pigments, and total phenols. Overall, the unknown components remained at a low level, further indicating that both the subcritical water method and the ultrasonic assisted extraction method have a high extraction selectivity for lily polysaccharides.

### Molecular weight of lily polysaccharides

It is well-known that the molecular weight of polysaccharides not only reflects the length of molecular chain, but also closely related to the physicochemical properties and biological activities of polysaccharides (37, 38). Therefore, the molecular weight of LP was determined by GPC technology in this paper (Table 2), and the results showed that there were multiple differences between the molecular weight of lily polysaccharides obtained by two extraction methods. The molecular weights of LP obtained by SW treatment ranged from 322 to 389 kDa. The molecular weight of LP extracted by UAE ranged from 3 to 53 kDa. The results showed that ultrasonic degradation of glycosidic bonds was stronger than that of subcritical water, resulting in the rupture of polysaccharides into smaller parts. In addition, for subcritical water extraction, the largest molecular weight was LLAP, whereas for ultrasonic extraction, the largest molecular weight was LBVP. The molecular weight of LDWP obtained by the two extraction methods was the lowest. In a word, molecular weight is closely related to the lily species and extraction method. Therefore, the appropriate species and extraction methods should be selected according to the actual production purposes.

### Monosaccharide composition

**FIGURE 2:**
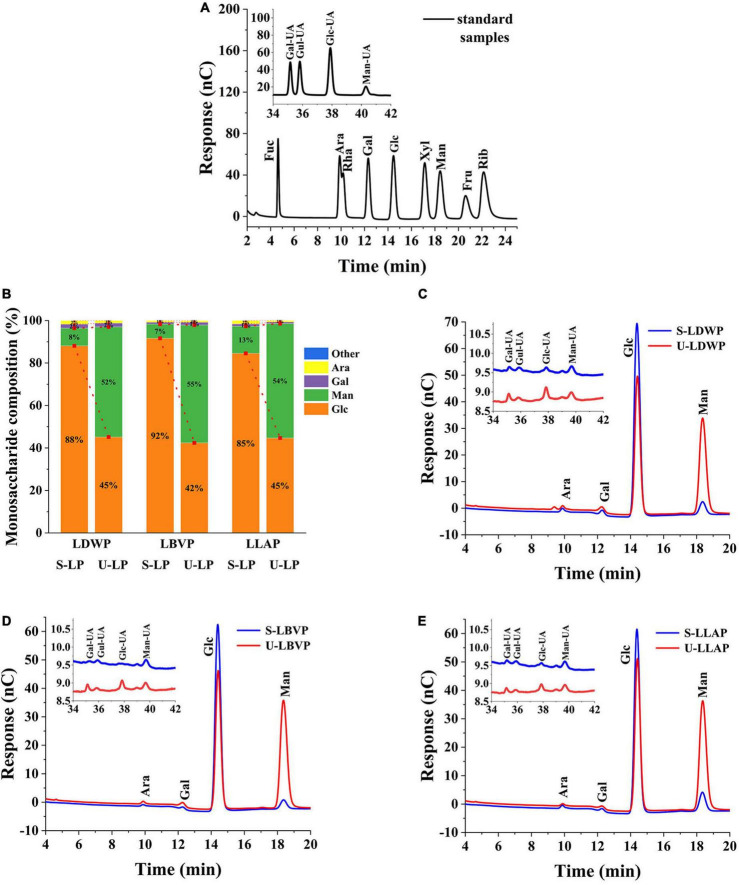
Diagram of HPAEC results. **(A)** Standard sample. **(B)** Monosaccharide ratio of all samples. **(C)** *Lilium davidii* var. *willmottiae* polysaccharides (LDWP) sample. **(D)** *Lilium brownii* var. *viridulum* polysaccharides (LBVP) sample. **(E)** *Lilium lancifolium* polysaccharides (LLAP) sample.

Polysaccharides consist of one or more monosaccharides linked by glycosidic bonds. According to the monosaccharide formation of polysaccharides, it can be divided into homopolysaccharides and heteropolysaccharides (39). As shown in the Figure 2, LP were heteropolysaccharides, which were mainly composed of glucose (Glc), mannose (Man), galactose (Gal), and arabinose (Ara) and contained a small amount of galacturonic Acid (Gal-UA), glucuronic Acid (Glc-UA), mannose Acid (Man-UA), and Guluronic Acid (Gul-UA). Specifically, compared with lily species, extraction methods had a greater effect on monosaccharide composition, as can be seen from the percentage of monosaccharides in all LP samples (Figure 2B). The Glc content in LP extracted from subcritical water was the highest. And the proportions of Glc in S-LDWP, S-LBVP and S-LLAP were 88.09, 91.67, and 84.57%, respectively. However, for UAE extracted LP, Glc and Man were the main components, the percentage of Glc in U-LP ranged from 42.41 to 45.16%, and the proportion of Man in U-LP ranged from 51.85 to 55.50%. The proportion of other monosaccharides in U-LP except Glc and Man is between 1 and 3%.

These results showed that different extraction methods had no significant effect on the monosaccharide types, but significantly affected the percentage and molar ratio of monosaccharide. Monosaccharide composition has been reported to affect the biological activity of polysaccharides to some extent (40, 41). Therefore, the extraction method and species can be selected according to the purpose of preparing the polysaccharides.

### Spectroscopic characterization of lily polysaccharides

#### Ultraviolet–visible spectrum

**FIGURE 3:**
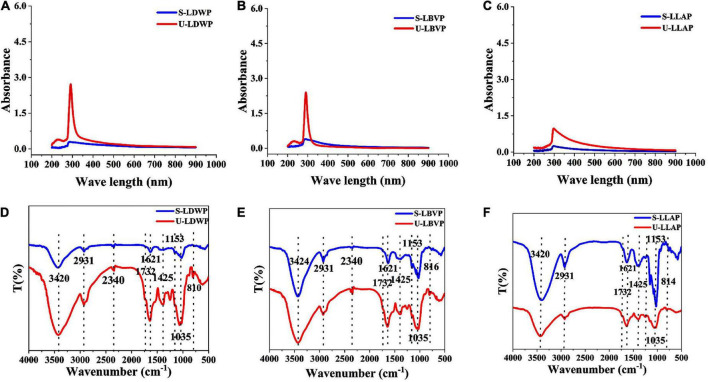
Spectrum properties of three lily polysaccharides extracted by different extraction methods. **(A–C)** The ultraviolet–visible (UV) analysis. **(D–F)** Fourier transform infrared (FT-IR) analysis.

UV absorption spectra of the series lily polysaccharides obtained by different extraction methods were shown in Figures 3A–C. All the LP samples showed weak absorption peaks only at 280 nm, which indicated that the content of non-polysaccharides with UV absorption in crude LP samples were low except protein. Moreover, it was obvious that the absorption peak of subcritical water extracted lily polysaccharides (S-LP) at 280 nm was much lower than that of U-LP, which is consistent with the previous results of protein content measured by coomassie brilliant blue. The results showed that subcritical water method could obtain polysaccharides with lower protein content.

#### Fourier transform infrared spectrum

Regardless of the extraction method and species, the absorption peaks of LP samples showed high similarity (Figures 3D–F). The broad peak at 3,420–3,424 cm^–1^ was attributed to the stretching vibration of O-H and the strong and broad peak shape was a typical band for carbohydrates, which indicated the presence of inter-molecular or intra-molecular hydrogen bonds (42). The peak at 2,931 cm^–1^ was caused by C-H stretching vibration of LP (43). Except for LLAP, LDWP, and LBVP had characteristic absorption peaks at 2,340 cm^–1^, indicating that amide groups exist in LDWP and LBVP. Moreover, the absorption peaks at 1,621 and 1,732 cm^–1^ were caused by the free carboxyl (COO^–^) groups and esterified carboxyl group (COO-CH_3_) in all polysaccharides (9). In addition, the peak at 1,425 cm^–1^ was also caused by CH_2_ vibration. 1,153 and 1,035 cm^–1^ correspond to the absorption vibrations of pyranose and Glc, respectively (44). In addition, the weak bands near 816 cm^–1^ may be compatible with the presence of α-D-mannose in LP samples (9).

### Scanning electron microscopy analysis of lily polysaccharides

**FIGURE 4:**
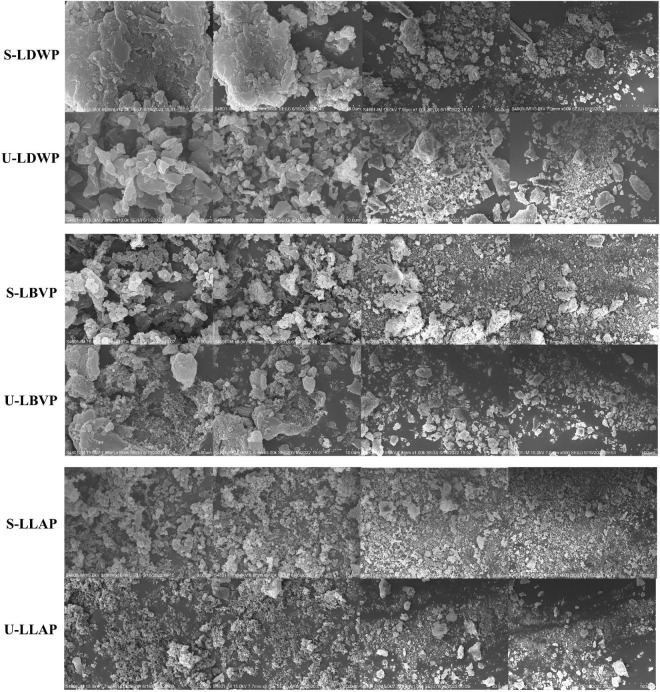
Scanning electron microscope (SEM) images of three lily polysaccharides extracted by different extraction methods at 5, 10, 50, and 100 μm.

Different micromorphology characteristics is one of the key factors leading to the complexity of polysaccharides (45). As shown in Figure 4, the surface morphology and structure of LP were affected by different extraction methods. Duan et al. obtained the same conclusion in the extraction of Lentinan (42). Although all polysaccharide samples showed agglomeration phenomenon, there were some differences in the micromorphology of LP between different species. In detail, LDWP samples had smooth surfaces and irregular properties. Moreover, both LBVP and LLAP samples showed dense clusters of quasi-spherical honeycomb particles, and LLAP samples had smaller particle sizes. Ji et al. speculated that the structural aggregation of polysaccharide molecules may be closely related to the presence of carboxyl and hydroxyl groups (37).

### Rheological characterization

**FIGURE 5:**
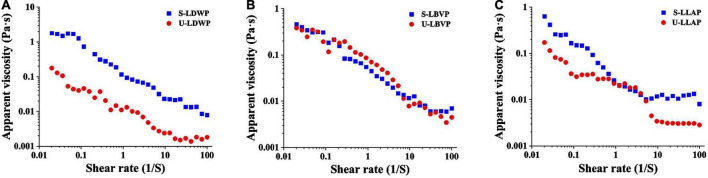
The flow behavior of three lily polysaccharides extracted by different extraction methods. **(A)** *Lilium davidii* var. *willmottiae* polysaccharides (LDWP), **(B)** *Lilium brownii* var. *viridulum* polysaccharides (LBVP), **(C)** *Lilium lancifolium* polysaccharides (LLAP).

Some natural polysaccharides have been widely used in the preparation of gels, thickeners and emulsifiers due to their excellent viscosity (46). As shown in Figure 5, with the increase of shear rate, the apparent viscosity of LP samples decreased significantly, and which showed typical shear-thinning behavior of Newtonian fluid or pseudoplastic fluid (33). The shear thinning behavior of polysaccharides may be related to the unwinding of molecular chains in solution (47). Interestingly, independent of species, both initial and final viscosity of S-LP samples were higher than those of U-LP at high shear rates. Polysaccharide samples extracted by subcritical water with high apparent viscosity are suitable for development as gels or thickeners.

## Conclusion

In conclusion, compared with lily species, the extraction method has a greater impact on the properties of LP. Subcritical water extraction could increase the polysaccharides yield by 4.52–14.88 times than ultrasound-assisted extraction. S-LP samples have high contents of neutral glucose and uronic acid. Ultrasound-assisted extraction was easier to obtain polysaccharides with higher reducing sugar content and lower molecular weight. Moreover, the extraction method also had a significant effect on the monosaccharide composition of polysaccharides. Glc was the key component in S-LP, while Glc and Man were the key components in U-LP. In addition, the rheological behavior of polysaccharides indicated that LDWP extracted from subcritical water had the largest apparent viscosity. Interestingly, S-LDWP samples had low solubility, making them suitable for development as gels, thickeners, and emulsifiers. Hence, the extraction method and lily species had a great influence on the physicochemical properties of lily polysaccharides, and the most suitable method and species should be selected according to the research purpose in practical production applications.

## Data availability statement

The original contributions presented in this study are included in the article/supplementary material, further inquiries can be directed to the corresponding authors.

## Author contributions

ZS: investigation, conceptualization, formal analysis, writing—original draft, and writing—review and editing. YZ: formal analysis, data curation, and writing—original draft. YL: investigation and visualization. YRT: investigation, methodology, and data curation. WW, YB, YCT, YH, and LX: investigation. JM: funding acquisition, supervision, and writing—review and editing. PY: investigation, funding acquisition, supervision, and writing—review and editing. All authors contributed to the article and approved the submitted version.

## Abbreviations

LP: Lily polysaccharides
S-LP: Subcritical water extracted lily polysaccharides
U-LP: Ultrasound-assisted extracted lily polysaccharides
LDWP: *Lilium davidii* var. *willmottiae* polysaccharides
LBVP: *Lilium brownii* var. *viridulum* polysaccharides
LLAP: *Lilium lancifolium* polysaccharides.
